# Thymol-Modified Oleic and Linoleic Acids Encapsulated in Polymeric Nanoparticles: Enhanced Bioactivity, Stability, and Biomedical Potential

**DOI:** 10.3390/polym16010072

**Published:** 2023-12-26

**Authors:** Maria B. Sokol, Vera A. Sokhraneva, Nataliya V. Groza, Mariia R. Mollaeva, Nikita G. Yabbarov, Margarita V. Chirkina, Anna A. Trufanova, Vladimir I. Popenko, Elena D. Nikolskaya

**Affiliations:** 1 N.M. Emanuel Institute of Biochemical Physics of Russian Academy of Sciences, 119334 Moscow, Russia; mollaeva-mr@sky.chph.ras.ru (M.R.M.); yabbarov_ng@sky.chph.ras.ru (N.G.Y.); chir.marg@sky.chph.ras.ru (M.V.C.); puzinoaa@gmail.com (A.A.T.); 2 N.A. Preobrazhensky Department of Chemistry and Technology of Biologically Active Compounds, Medicinal and Organic Chemistry, M.V. Lomonosov Institute of Fine Chemical Technologies, MIREA—Russian Technological University, 119571 Moscow, Russia; sokhraneva.v@mail.ru (V.A.S.); grozanv@gmail.com (N.V.G.); 3 Engelhardt Institute of Molecular Biology, Russian Academy of Sciences, 11999 Moscow, Russia; popenko@eimb.ru

**Keywords:** oleic acid, linoleic acid, biological activity, PLGA, nanoparticles

## Abstract

Unsaturated fatty acids, such as oleic acid (OA) and linoleic acid (LA), are promising antimicrobial and cytostatic agents. We modified OA and LA with thymol (TOA and TLA, respectively) to expand their bioavailability, stability, and possible applications, and encapsulated these derivatives in polymeric nanoparticles (TOA-NPs and TLA-NPs, respectively). Prior to synthesis, we performed mathematical simulations with PASS and ADMETlab 2.0 to predict the biological activity and pharmacokinetics of TOA and TLA. TOA and TLA were synthesized via esterification in the presence of catalysts. Next, we formulated nanoparticles using the single-emulsion solvent evaporation technique. We applied dynamic light scattering, Uv-vis spectroscopy, release studies under gastrointestinal (pH 1.2–6.8) and blood environment simulation conditions (pH 7.4), and in vitro biological activity testing to characterize the nanoparticles. PASS revealed that TOA and TLA have antimicrobial and anticancer therapeutic potential. ADMETlab 2.0 provided a rationale for TOA and TLA encapsulation. The nanoparticles had an average size of 212–227 nm, with a high encapsulation efficiency (71–93%), and released TOA and TLA in a gradual and prolonged mode. TLA-NPs possessed higher antibacterial activity against *B. cereus* and *S. aureus* and pronounced cytotoxic activity against MCF-7, K562, and A549 cell lines compared to TOA-NPs. Our findings expand the biomedical application of fatty acids and provide a basis for further in vivo evaluation of designed derivatives and formulations.

## 1. Introduction

Oleic acid (OA) and linoleic acid (LA) are unsaturated fatty acids (FAs)—essential parts of cell membranes and crucial players in various physiological processes. In recent years, these fatty acids have gathered significant attention due to their potential antibacterial and anticancer properties.

Various studies have highlighted the antibacterial potential of OA and LA against a broad spectrum of pathogens. These fatty acids exhibited direct antimicrobial activity, disrupting microbial membranes and hindering essential cellular processes [1]. Several studies demonstrated the high efficacy of OA and LA against prominent hospital pathogens, including *Staphylococcus aureus* [2], *Helicobacter pylori* [3], and various *Salmonella* strains [4].

Apart from their antibacterial properties, OA and LA have also demonstrated potential anticancer activity. These FAs suppressed cancer cell proliferation, induced apoptosis, inhibited angiogenesis, and modulated signaling pathways implicated in tumor growth and metastasis formation [5,6]. OA and LA were effective against various cancer types, including breast, prostate, lung, and colon cancer [7]. 

Recent studies addressed the stability improvement in body fluids [8], the cell penetration enhancement [9], and the capacity for synergetic improvement of biological properties of FAs conjugated with phenolic compounds [10,11].

Thus, we proposed that conjugates of OA and LA with phenolic compounds such as thymol (2-isopropyl-5-methylphenol) would result in an increase in the biological effects of both compounds. Thymol is applied widely as an antimicrobial or cytostatic agent and has a natural origin [12,13].

The highly lipophilic nature of both FAs and thymol resulted in the hindered efficacy, bioavailability, and applicability of the conjugates. Thus, we applied the strategy of drug loading into nanoparticles to address these issues. Among numerous matrixes for drug delivery and nanoparticle formulation, we selected poly(lactic-co-glycolic acid) (PLGA) due to its relatively fast biodegradability and high biocompatibility [14]. Moreover, many regulators, including the FDA and the EMA, have already approved PLGA-based drugs for medical application [15]. Among the most important advantages of PLGA application, we can emphasize the following: support of the properties and structures of cytostatic and antimicrobial molecules loaded into matrixes [16,17]; the reproducible in silico simulation of drug–polymer interaction results [18]; the effective encapsulation of lipophilic compounds [19]; and the simplified application and administration of resultant PLGA formulations due to their high stability and water solubility [20].

In the present study, we applied computational methods to evaluate the range of potential activities of conjugates of OA and LA with thymol. Next, we described, for the first time, the one-batch synthesis of thymol-conjugated OA (TOA) and LA (TLA). Further, we designed novel TOA- and TLA-loaded PLGA nanoparticles (TOA-NPs and TLA-NPs, respectively) and analyzed the physico-chemical properties of the resultant formulations. Finally, we performed antimicrobial testing of the TOA-NPs and TLA-NPs against *E. coli*, *B. cereus*, *S. aureus*, *C. albicans*, and *A. niger* 37a strains and an anticancer activity evaluation against human non-small-cell lung carcinoma A549, human breast adenocarcinoma MCF-7, and human myelogenous leukemia K562.

## 2. Materials and Methods

### 2.1. Materials

Thymol (99% purity), oleic acid (OA) (≥99% purity), linoleic acid (LA) (≥99% purity), and doxorubicin hydrochloride (≥99% purity) were purchased from Sigma-Aldrich (St. Louis, MO, USA). Dimethyl sulfoxide (DMSO), petroleum ether (PE), ethyl acetate (EA), methylene chloride, and acetone were purchased from Ruskhim (Moscow, Russia). The solvents were used as extra-pure grade. Water was purified in a Millipore Milli-Q Plus system (Darmstadt, Germany). N,N-dicyclohexylcarbodiimide (DCC) and 4-dimethylaminopyridine (DMAP) were obtained from Sigma-Aldrich (St. Louis, MO, USA). Silica gel Kieselgel 60 with a particle size of 60–100 µm was obtained from Мerck (Darmstadt, Germany). NaCl, HCl, NaOH, KH_2_PO_4_, K_2_CO_3_, NaHCO_3_, СаН_2_, and KOH were purchased from KhimMed (Moscow, Russia). Deuterochloroform CDCl_3_ was obtained from Sigma-Aldrich (St. Louis, MO, USA).

Poly(d,l-lactide-co-glycolide) (PLGA polymer with carboxylic terminal group, 50/50, of inherent viscosity, 0.2 dL/g in HFIP, MW 17,000–21,000), was purchased from LACTEL Absorbance Polymers (Birmingham, AL, USA). D-mannitol, Koliphore P388, and polyvinyl alcohol (PVA, MW 30,000–70,000, 87–90% hydrolyzed) were purchased from Sigma-Aldrich (St. Louis, MO, USA). Phosphate-buffered saline (PBS) was purchased from Amreso (Solon, OH, USA).

Sabouraud dextrose agar was purchased from Becton Dickinson (Heidelberg, Germany). Mueller–Hinton Broth and CASO agar medium were purchased from Sifin (Berlin, Germany).

DMEM and RPMI 1640 culture media were purchased from Gibco (Waltham, MA, USA). 3-(4,5-dimethyl-thiazol-2yl)-2,5-diphenyltetrazoliumbromide (MTT) was purchased from Sigma-Aldrich (St. Louis, MO, USA). Fetal bovine serum (FBS) was purchased from Hyclone (Logan, UT, USA).

### 2.2. PASS-Based In Silico Assessment of the Biological Activity of the Modified Fatty Acids

The prediction of possible antitumor activity was assisted with the PASS online service [21]. The compound structures were loaded as SMILES files generated with the molecular-structure drawing software ACD/ChemSketch.

### 2.3. ADMET Simulations of the Modified Fatty Acids

In silico evaluation of the pharmacokinetic profiles and toxicity of TOA and TLA was performed with ADMETlab 2.0 (https://admetmesh.scbdd.com/pub/ (accessed on 22 August 2022)).

### 2.4. Synthesis of TOA and TLA

The compounds were purified using adsorption chromatography with a silica gel Kieselgel as a stationary phase. The substances were analyzed by thin-layer chromatography (TLC) on SilicaGel 60 F254 plates (Merck, Darmstadt, Germany) with subsequent detection in phosphoromolybdic acid alcohol solution.

^1^H-NMR and ^13^C-NMR spectra were recorded on an MSL-300 pulse Fourier spectrometer (Brucker, Germany) with an operating frequency of 300 MHz. The samples were dissolved in deuterochloroform CDCl_3_. Tetramethylsilane was used as an internal standard for ^1^H-NMR, and the deuterium signal in CDCl_3_ was used for ^13^C-NMR.

TOA and TLA compositions were determined by elemental analysis using a CHNSO Flash EA 1112 analyzer (Thermo Finnigan, Italy).

#### 2.4.1. Synthesis of TOA

Oleic acid (2.124 mmol, 600 mg) was dissolved in 5 mL CH_2_Cl_2_ and transferred to a flask with a magnetic stirrer. DCC (1.912 mmol, 394.5 mg) was added to the acid solution, and the mixture was stirred for 15 min at room temperature to form oleic anhydride. Then, thymol (2.124 mmol, 319.1 mg) was added to the reaction mixture in the presence of a catalyst, DMAP (0.646 mmol, 78.9 mg). The reaction mixture was continuously stirred for 20 h in the dark. Then, the reaction mixture was filtered, and the filtrate was evaporated using a rotary evaporator. The progress of the reaction was monitored by TLC using a solvent system with petroleum ether (PE) and ethyl acetate (EA): PE/EA, in a ratio of 2:1. The mixture was purified by column chromatography (PE/EA, 9:1). Yield (61%), Rf = 0.72 (PE/EA, 2:1). 

^1^H NMR (300 MHz, CDCl_3_) (Figure S1): δ = 0.88 (-СН_3_, 3H), 1.19 (Ar-CH-(CH_3_)_2_, 6H), 1.25–1.60 (10 -СН_2_-, 20H), 1.78 (-СОСН_2_-СН_2_-, 2H), 2.03 (2 -СН_2_-СН=, 4H), 2.31 (Ar-CH_3_, 3H), 2.57 (-COCH_2_-, 2H), 2.97 (Ar-CH-(CH_3_)_2_, 1H), 5.36 (-CH=CH-, 2H), 6.80 (Ar-H_5_, 1H), 7.03 (Ar-H_4_, 1H), 7.18 (Ar-H_2_, 1H).

^13^C NMR (75 MHz, CDCl_3_) (Figure S3): δ = 14.2, 21, 22.7–23.1, 25.2, 27.2, 27.5, 29.1–30, 31.9–32.1, 34.6, 122.9, 126.2, 127.2, 129.7, 130.2, 136.7, 137.5, 148.0, 172.5.

Elemental analysis (%): calc. for С_28_Н_46_О_2_: C 81.10, H 11.18; found: C 81.27, H 10.93.

#### 2.4.2. Synthesis of TLA

Linoleic acid (2.139 mmol, 600 mg) was dissolved in 5 mL CH_2_Cl_2_ and transferred to a flask with a magnetic stirrer. DCC (1.925 mmol, 397.1 mg) was added to the acid solution, and the mixture was stirred for 15 min at room temperature to form linoleic anhydride. Then, thymol (2.139 mmol, 321.3 mg) was added to the reaction mixture in the presence of the catalyst, DMAP (0.65 mmol, 79.4 mg). The reaction mixture was continuously stirred for 20 h in the dark. Then, the reaction mixture was filtered, and the filtrate was evaporated using a rotary evaporator. The progress of the reaction was monitored by TLC using a solvent system: PE/EA, in a ratio of 2:1. The mixture was purified by column chromatography (PE/EA, 9:1). Yield (45%), Rf = 0.93 (PE/EA, 2:1).

^1^H NMR (300 MHz, CDCl_3_) (Figure S2): δ = 0.31 (-СН_3_, 3H), 1.21 (Ar-CH-(CH_3_)_2_, 6H), 1.32–1.39 (7 -СН_2_-, 14H), 1.81 (-СОСН_2_-CH_2_, 2H), 2.08 (2 -СН_2_-СН=, 4H), 2.32 (Ar-CH_3_, 3H), 2.61 (-COCH_2_-, 2H), 2.81 (=СН-СН_2_-СН=, 2H), 2.98 (Ar-CH-(CH_3_)_2_, 1H), 5.39 (2 -CH=CH-, 4H), 6.80 (Ar-H_5_, 1H), 7.04 (Ar-H_4_, 1H), 7.19 (Ar-H_2_, 1H).

^13^С NMR (75 MHz, CDCl_3_) (Figure S4): δ = 14.21, 20.96, 22.72, 23.16, 25.18, 25.78, 27.20 × 2, 27.34, 29.30–31.67, 34.35, 37.42, 122.87, 126.47, 127.14, 128.03, 128.22, 130.13, 130.35, 136.63, 137.12, 148.06, 172.6.

Elemental analysis (%): calc. for С_28_Н_44_О_2_: C 81.49, H 10.74; found: C 81.68, H 10.56.

### 2.5. NPs Loaded with OA, LA, TOA, and TLA Formulations

PLGA NPs loaded with the conjugates and with the unmodified fatty acids were formulated by the single-emulsion solvent evaporation technique. A quantity of 500 mg of compound was dissolved in 1 mL of CH_2_Cl_2_, and 50 mg of PLGA was dissolved in 5 mL of CH_2_Cl_2_. Then, 20 µL of substance solution was transferred to the PLGA solution. The resultant solution was added dropwise to 25 mL of 1% *w*/*v* PVA aqueous solution and stirred for 10 min. Next, an emulsion was formed by ultrasonic homogenization (Labsonic U. B. Braun, Germany) repeated three times for 35 s at 25 s intervals in an ice bath. The organic solvent was then rapidly removed with a vacuum rotary evaporator (IKA HB10, Staufen, Germany) at +30 °C. The obtained sample was separated by centrifugation for 30 min at 14,000 rpm and +4 °C (Beckman J221, Brea, CA, USA). The supernatant was decanted, and deionized water was poured into the centrifuge tube, followed by vigorous stirring to wash the NP pellets. Finally, 10% (*w*/*v*) D-mannitol was added, and the mixture was freeze-dried by lyophilization (Alpha 2–4, Martin Christ GmbH, Osterode am Harz, Germany). 

The scale-up of NPs loaded with OA, LA, TOA, and TLA was similar to the standard lab-scale preparation described above, and the original ratios between the components were retained. To produce 4-fold (8- or 12-fold) scaled-up batches, 4 volumes (8 or 12 volumes) of CH_2_Cl_2_ in different vessels, each containing FAs (500 mg/mL) and PLGA (10 mg/mL), were mixed and added dropwise to corresponding quantities of 1% PVA aqueous solution. The suspensions were homogenized, and the resultant emulsions were mixed in one vessel. The organic solvent evaporation and mixture lyophilization were performed as described above.

Blank NPs were synthesized as described above, except for substance addition.

### 2.6. Characterization of the NPs Loaded with OA, LA, TOA, and TLA

#### 2.6.1. Size, ζ-Potential, and Polydispersity Index Measurement

Particle sizes, zeta-potentials, and polydispersity indexes (PDIs) were determined with a Ζeta-sizer Nano ZS ZEN 3600 analyzer (Malvern Instruments, Malvern, UK) as described previously [20]. The responses were measured in triplicate.

#### 2.6.2. Drug Loading (DL) and Entrapment Efficiency (EE) Measurement

The DL of the obtained nanoparticles was determined by a UV-vis spectrophotometer, the Spectronic Helios Alpha (Thermo Fisher Scientific, Waltham, MA, USA); 8 mg of lyophilized NPs was dissolved in 4 mL of DMSO, and, subsequently, absorption analysis was performed at 267 nm. The DL of the obtained nanoparticles was calculated according to Equation (1):

DL, % = (weight of remained drug in the particles/weight of particles) × 100(1)

To determine EE, 8 mg of NPs was dissolved in 2 mL of distilled water and centrifuged at 14,000 rpm for 15 min at +4 °C. The supernatant was withdrawn, and a pellet was dissolved in 4 mL of DMSO. Absorption analysis was carried out at 267 nm. EE was calculated according to Equation (2):

EE, % = 100 − ((total amount of drug − actual amount of drug in precipitate)/ total amount of drug)) × 100(2)

#### 2.6.3. Fourier Transform Infrared Spectroscopy (FTIR)

IR spectra were recorded on an EQUINOX 55 Fourier spectrometer (Brucker, Billerica, MA, USA) in the wavenumber range from 400 to 4000 cm^–1^. Samples were prepared using dichloromethane (for substances and PLGA) and the powder method in tablets with KBr (for nanoparticles).

#### 2.6.4. Morphological Study

The shape and surface morphologies of the NPs were examined with transmission electron microscopy (TEM). NP suspension in distilled water (1 mg/mL) was applied to freshly ionized coal-formed films, and the sample was contrasted with 1% uranium acetate aqueous solution. The visualization was performed using a 100СХ microscope (JEOL, Tokyo, Japan) at an accelerating voltage of 80 kV.

### 2.7. In Vitro Drug Release of OA, LA, TOA, and TLA from NPs

#### 2.7.1. pH 1.2–6.8 for Oral Administration (Antimicrobial Activity)

A quantity of 70 mg of NPs was dispersed in simulated gastric fluid (200 mg NaCl, 8 mL 1 M HCl, 100 mL water, pH 1.2) with 0.1% *w*/*v* Koliphore. For each sample, the solution was divided into 12 microcentrifuge tubes with continuous shaking at 120 rpm at +37 °C. The tubes were withdrawn and centrifuged at 3000 rpm for 15 min after predetermined time intervals (0, 0.25, 0.5, 1, 1.5, 2, 3, 4, 4.25, 5, 5.5, and 24 h). After 4 h of incubation, the simulated gastric fluid was replaced with simulated intestinal fluid (620 mg NaOH, 680 mg KH_2_PO_4_, 100 mL water, pH 6.8). After removing the supernatant, the pellet was resuspended with 0.75 mL of DMSO for UV-vis detection at 267 nm.

#### 2.7.2. pH 7.4 for Parenteral Administration (Cytotoxic Activity)

In vitro OA, LA, TOA, and TLA release from the NPs was carried out at +37 °C in 0.01M PBS (pH 7.4). Plastic tubes (4 NP batches in triplicate; safe-lock tubes, 2.0 mL, Eppendorf, Hamburg, Germany) were filled with 12 mg of lyophilized NPs and diluted with 2 mL of 0.01 M PBS. The tubes were placed into a horizontal shaker (+37 °C, 100 rpm, MS 3 basic; IKA, Staufen, Germany). At predetermined time points, the samples were withdrawn, centrifuged at 5000× *g* for 5 min at room temperature (5417R, Eppendorf, Hamburg, Germany), and the resultant pellet was freeze-dried. The samples were analyzed in triplicate. The amounts of residual substances in the NPs were determined by UV-vis spectrophotometry using a previously described protocol.

The mathematical modeling of OA, LA, TOA, and TLA release profiles from NPs was performed with the DDSolver^®^ add-in program for Microsoft Excel.

### 2.8. Antibacterial and Antifungal Efficacy Test

#### 2.8.1. Cell Culture

All microbial strains, including three bacterial strains, *Staphylococcus aureus* (*S. aureus*, ATCC 25923), *Escherichia coli* (*E. coli*, ATCC 25922), and *Bacillus cereus* (*B. cereus* ATCC 10702); a yeast strain, *Candida albicans* (*C. albicans* ATCC 10231); and a mold strain, *Aspergillus niger* (*A. niger* ATCC 16404), were provided by the Russian National Collection of Industrial Microorganisms.

The microorganisms were kept at −75 °C in casein–soymeal–peptone broth with 10–15% glycerol. Before the experiment, a bacterial strain was activated from cryoconservation by seeding it onto CASO Agar medium and incubated at +35 ± 2 °C for 18–20 h. *Candida albicans* was seeded onto Sabouraud Dextrose Agar medium and incubated at +35 ± 2 °C for 48 h, and *A. niger* was incubated for 5–7 days until the formation of sporulating aerial mycelia. The spores of the *A. niger* were collected and mixed with sterile buffer solution to make a spore suspension.

Overnight bacterial strains were seeded onto Mueller–Hinton Broth and adjusted to have 0.5 McFarland standard turbidity. Microorganism suspension turbidity was determined by using a DEN-1 densitometer (suspension turbidity detector; BioSan, Riga, Latvia).

The microorganisms were incubated further at +35 ± 2 °C to obtain 1 McF optical turbidity. Then, they were precipitated by centrifugation and washed with phosphate-buffered saline (PBS). The cell densities were adjusted with PBS to reach 0.5 McF and were equal to ca. 1.5 × 10^8^ CFU per mL (cells per mL).

#### 2.8.2. Preparation of the Test Samples

Thymol, OA, LA, TOA, and TLA were dissolved in 0.5 mL of DMSO, followed by dilution with 1 mL of Mueller–Hinton broth to a concentration of 30,000 µg/mL. NPs loaded with OA, LA, TOA, and TLA were diluted with Mueller–Hinton broth to free PUFA equivalent concentrations.

#### 2.8.3. Determination of Minimum Inhibitory Concentration (MIC) and Minimum Bactericidal Concentration (MBC)

The MIC (the lowest concentration of a drug that inhibits the visible growth of bacteria) of thymol, NPs, and free OA, LA, TOA, and TLA was determined using a broth microdilution assay [22]. Briefly, 50 µL (for bacterial strains) or 100 µL (for *C. albicans* and *A. Niger*) of the test samples were added to the first wells of 96-well plates. The concentration range was from 1500 µg/mL to 7.3 µg/mL. Inoculated plates were incubated for 24 h (bacterial strains), 48 h (*C. albicans*), and 72 h (*A. Niger*) at +32.5 ± 2.5 °C.

A set of control wells was prepared: (1) positive control wells: broth medium inoculated with the tested microorganisms; (2) negative control wells: sterile broth medium in the absence of NPs, drugs, and microorganisms; (3) blank NPs at a corresponding amount of NPs loaded with OA, LA, TOA, and TLA. Each sample/control was prepared in triplicate.

The lowest concentration of a test sample with no visual turbidity in the plate well was considered the MIC. Next, 10 µL of clear solution from the plate well was dropped on an agar plate, and the plate was incubated for 24 h at +32.5 ± 2.5 °C. The minimum concentration of a test sample with no colonies present on the agar was considered the MBC.

### 2.9. Cytotoxic Activity Test

#### 2.9.1. Cell Culture

A549 (human non-small-cell lung carcinoma) and MCF-7 (human breast adenocarcinoma) (ATCC, The Global Bioresource Center, Manassas ,VA, USA) cells were maintained in Dulbecco’s modified Eagle’s medium (DMEM), and K562 (human myelogenous leukemia) (ATCC, The Global Bioresource Center, USA) cells were maintained in RPMI 1640 supplemented with 10% fetal bovine serum and gentamycin (50 µg/mL). The cells were grown in plastic 25 cm^2^ cell culture flasks at +37 °C in a humidified atmosphere containing 5% CO_2_ (Sanyo, Osaka, Japan). The cells were seeded before reaching 80% confluence by detachment with trypsin/EDTA solution.

#### 2.9.2. Cytotoxic Activity

A549 and MCF-7 cells were seeded in 96-well plates (5000 cells per well) 24 h before the experiment and incubated under standard conditions. K562 cells were seeded into wells (5000 cells per well) just after free OA, LA, TOA, and TLA or corresponding NPs were added into 96-well plates. All samples were added in triplets in the concentration range 0.01–2 mM (according to the concentration of substances) and then incubated for 72 h. Cell survival was determined using the standard MTT assay [23]: 50 µL MTT in DMEM/RPMI 1640 (1 mg/mL) was added into each well. After cell incubation for +37 °C, the medium was removed and precipitated formazan crystals were dissolved in 100 µL DMSO. Following this, the absorption intensity of formazan was measured at 540 nm on a microplate reader. Cell viability was determined as the percent of untreated control. The responses were measured in triplicate.

### 2.10. Statistical Analysis

Statistical analysis was performed using an independent-sample *t*-test with OriginPro 7.5 software. Differences between groups were considered significant at *p* ≤ 0.05.

## 3. Results and Discussion

### 3.1. PASS-Based In Silico Assessment of the Biological Activity of the Modified Fatty Acids

We used the PASS online service to predict the growth inhibition spectra of TOA and TLA against bacteria and fungi at concentrations below 10,000 nM. PASS expresses the score for each compound as an activity confidence. A high confidence means a higher chance of the predicted activity being true.

Antibacterial activity prediction revealed that conjugation of thymol with oleic and linoleic acids may potentially lead to an increase in activity against Gram-negative bacteria (Table 1). TOA and TLA exhibited a high confidence in activity against *Bacteroides stercoris*, while OA and LA lacked any activity against Gram-negative strains. Regarding Gram-positive bacteria, such as *Streptococcus viridans*, *Staphylococcus simulans*, and *Clostridium cadaveris*, the activity of unmodified OA and LA differed negligibly from that of TOA and TLA. Notably, TOA and TLA revealed lower activity confidences against *L. plantarum*—probiotic bacteria [24].

The antifungal activity confidence of the substances was in the 0.1–0.4 range, indicating low probable activity. The highest activity confidence of the synthesized conjugates predicted was against *Candida rugosa*, with a confidence level of 0.126 for TOA and one of 0.120 for TLA. *Candida rugosa* causes gastrointestinal tract (GIT) infection in some regions, exhibiting decreased susceptibility to polyenes and fluconazole [25].

Next, we analyzed the probability of anticancer activity of the compounds—active (Pa) or inactive (Pi). Pa > Pi indicates possible activity of a compound [26]. Table 2 shows the results of the anticancer activity prediction.

Both the conjugates and the substances exhibited pronounced anticancer activity. TOA and TLA retained intact FA activity against skin and lung cancer. 

Thus, based on PASS predictions, we concluded that TOA and TLA have a therapeutic potential against both bacteria and fungi, as well as cancer cell lines.

### 3.2. ADME Simulations of the Modified Fatty Acids

The initial assessment of a compound’s potential as a drug is a critical step in the development process. We predicted the ADME (absorption, distribution, metabolism, and excretion) of the modified FAs via ADMETlab 2.0 to describe their pharmacokinetic (PK) profiles. Table 3 shows the main parameters influencing drug PK and bioavailability.

We considered oral bioavailability (F30%) as the crucial parameter of absorption for orally administered drugs. The calculated results revealed that the tested compounds exhibited F30% values of 0.9–1, indicating poor oral bioavailability.

Next, we evaluated plasma protein binding (PPB) and volume of distribution (Vd) to describe the distribution behavior of the tested compounds. A high PPB level means a low probability of drug escape out of the blood vessels, decreasing the drug distribution among tissues. The simulation revealed that all the compounds exhibited PPB values > 90%, indicating high levels of plasma protein binding and probable low therapeutic indexes. On the other hand, especially in the case of the conjugates, high PPB levels were accompanied by relatively high Vd values—a parameter characterizing the duration of drug retention in plasma or redistribution into other tissues [27]. Vd influences the half-life and duration of a compound’s activity in a steady state [28]. An appropriate potential drug Vd value falls within the range of 0.04–20.00 L/kg. In our case, all the compounds exhibited Vd values in the range of 0.60–2.70 L/kg.

Regarding metabolism, one of the most important metabolizing enzyme systems is cytochrome P450 (CYP) [29]. ADMETlab 2.0 prediction revealed that the tested compounds are substrates of the CYP2C9 isoform, which had the highest metabolic activity in liver hepatocytes and kidney cells [30]—in some cases, it may cause drug–drug interactions.

Finally, we evaluated the half-lives (T1/2) of the compounds to assess their excretion rates (Table 3). Drugs with T1/2 values close to 1 are characterized by a long half-life (>3 h); T1/2 values close to 0 mean a shorter half-life (<3 h). The conjugation revealed the tendency for half-life value reduction in the following order: LA > OA > TLA > TOA.

Besides PK parameters, Table 3 contains the physico-chemical properties of molecules, which are important for drug–polymer interaction and binding efficacy prediction. The OA and LA modification increased the molecular weight and logP of TOA and TLA. The TOA and TLA structure flexibility values were lower than the values for OA and LA due to the modification of linear alkyl fatty acid residues with the phenolic planar structure. Despite the flexibility decrease, the values of the three described parameters were suitable for efficient drug encapsulation. They indicated strong interaction of the drug with hydrophobic (lactic) moieties of the PLGA and corresponded to effective permeation through the highly entangling and viscous polymer matrix [18].

Overall, our analysis provided valuable insights into the PK properties of the compounds. Despite PASS prediction of antimicrobial and anticancer activity, the poor oral bioavailability, the high levels of PPB, and the short half-lives could limit the therapeutic activity of the tested compounds. Based on our previous findings [20,31], we proposed to encapsulate the compounds into PLGA nanoparticles to avoid these limitations. We also concluded that the molecular weight, flexibility, and logP values of the modified OA and LA are suitable for their effective encapsulation.

### 3.3. Synthesis of TOA and TLA

**Figure 1:**
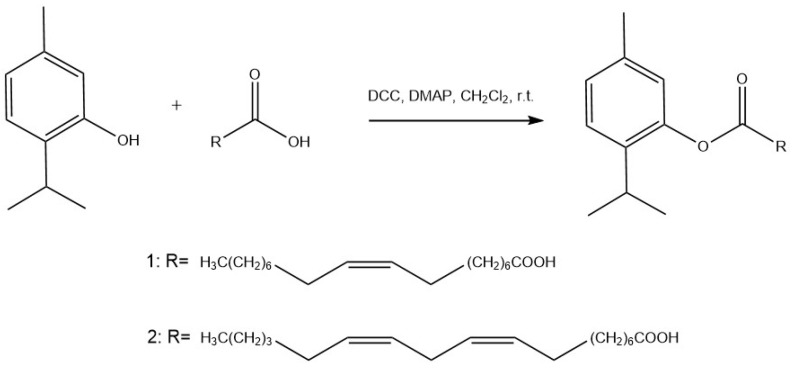
Synthesis of thymol-conjugated oleic acid **1** (2-isopropyl-5-methylphenyloleate; TOA) and **2** linoleic acid (2-isopropyl-5-methylphenyllinoleate; TLA).

We synthesized TOA and TLA by esterification of the hydroxyl group of a substituted phenol with a carboxyl group of a fatty acid in the presence of catalysts—N,N-dicyclohexylcarbodiimide (DCC) and 4-dimethylaminopyridine (DMAP)—as described previously [32]. Figure 1 describes the synthesis scheme for TOA and TLA. 

### 3.4. Formulation of the Nanoparticles

**Figure 2:**
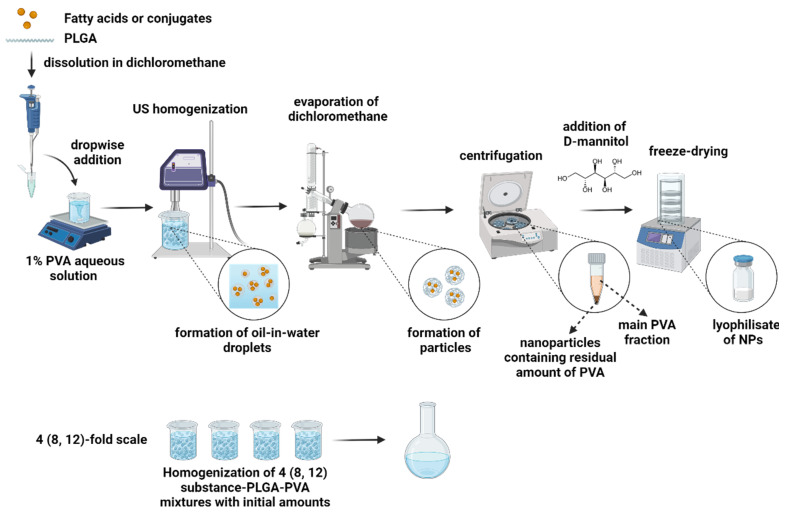
Formulation and scaling-up scheme for PLGA NPs loaded with OA, LA, TOA, and TLA.

We applied a single-emulsion solvent evaporation method to formulate PLGA nanoparticles loaded with OA, LA, TOA, and TLA. Both the PLGA and the compounds were dissolved in the oil phase, while PVA was presented in the aqueous phase as an emulsion stabilizer. After emulsification, we evaporated the organic solvent, triggering the precipitation of nanoparticles (Figure 2).

We evaluated the quality of the formulated NPs by particle size, PDI, zeta-potential, DL, and EE (Table 4) analyses. All samples exhibited sizes in the range of 207–282 nm and PDI values in the range of 0.099–0.289. These results corresponded to a monodispersed distribution of NPs and indicated suitable sizes for drug delivery via EPR effects [33]. The zeta-potential varied from −7.2 mV to −17.6 mV, which was sufficient for the colloidal stability of the NPs. The DL values for all samples were relatively high and varied from 8.0% to 28.1%, probably due to the effective interaction of FA residues with the polymer matrix and physical sorption in porous particles. EE values were in the range of 58–88%, with an unexpectedly low EE value for OA of 14%.

Further scaling-up of the NP formulations resulted in slight decreases in DL and EE values compared to the initial batches, probably due to the pronounced interaction of intact and modified FAs with PVA and their migration to the particle surfaces [34]. We observed a lack of significant influence on size and PDI values, which confirmed the efficacy of the NP formulation process. We selected 4-fold scaling for OA-NPs and LA-NPs and 12-fold scaling for TOA-NPs and TLA-NPs to maintain high EE values. 

**Figure 3:**
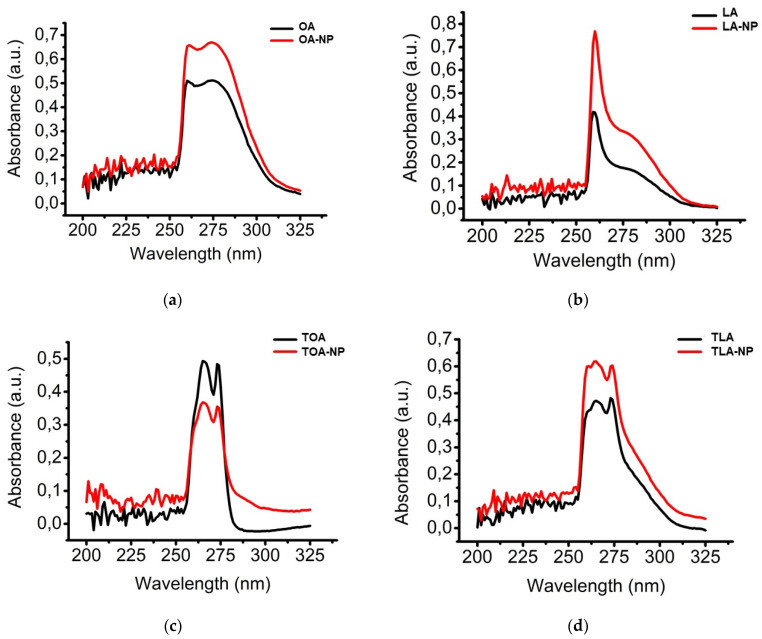
UV-vis spectra of (**a**) OA and OA-NPs, (**b**) LA and LA-NPs, (**c**) TOA and TOA-NPs, and (**d**) TLA and TLA-NPs.

We confirmed the stability of the intact and modified FAs after encapsulation by UV-vis spectroscopy (Figure 3). The UV-vis spectra of intact and modified FAs and the corresponding NPs had similar patterns, lacking new peaks of degradation products or peak shifts. Our results indicated that all compounds remained stable after encapsulation.

### 3.5. Morphological Analysis

**Figure 4:**
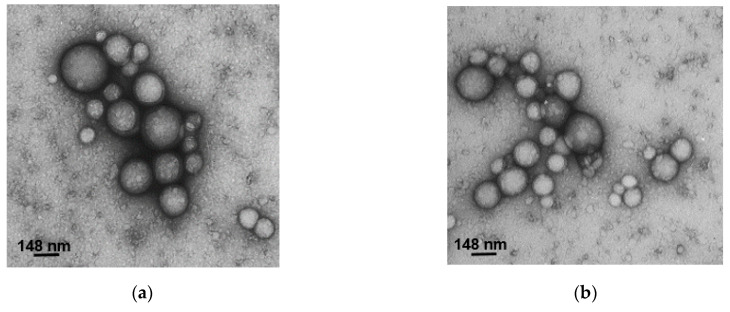

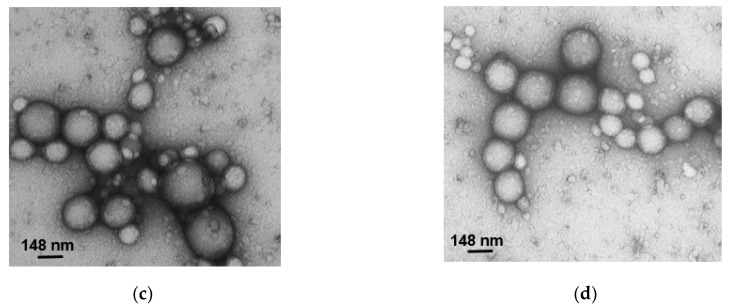
TEM images of (**a**) LA-NPs, (**b**) OA-NPs, (**c**) TLA-NPs, and (**d**) TOA-NPs.

We performed morphological analysis of the formulated NPs by TEM (Figure 4). All the samples were sphere-shaped and showed a smooth surface with a dispersion of sizes. The sizes observed by TEM were smaller than the sizes observed by DLS due to technique differences: during TEM, we saw the gyration radii of dried NPs, while during DLS we observed hydrodynamic diameters in suspension [35].

### 3.6. Fourier Transform Infrared Spectroscopy

**Figure 5:**
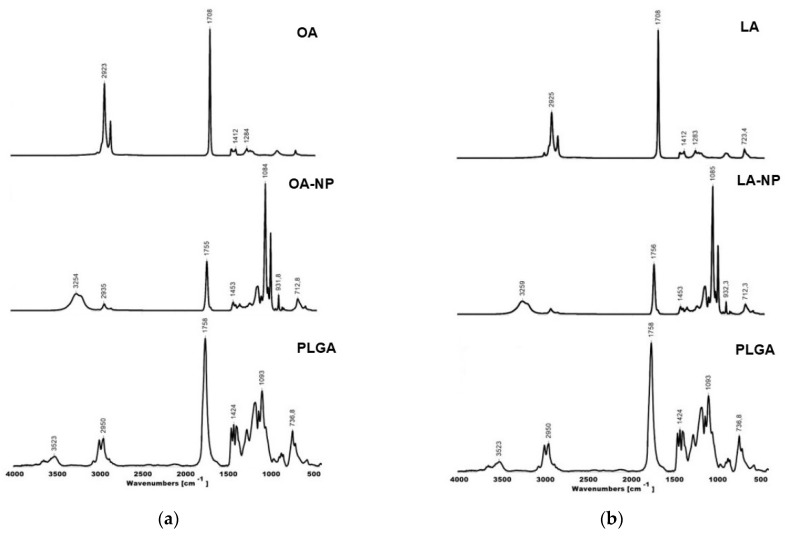

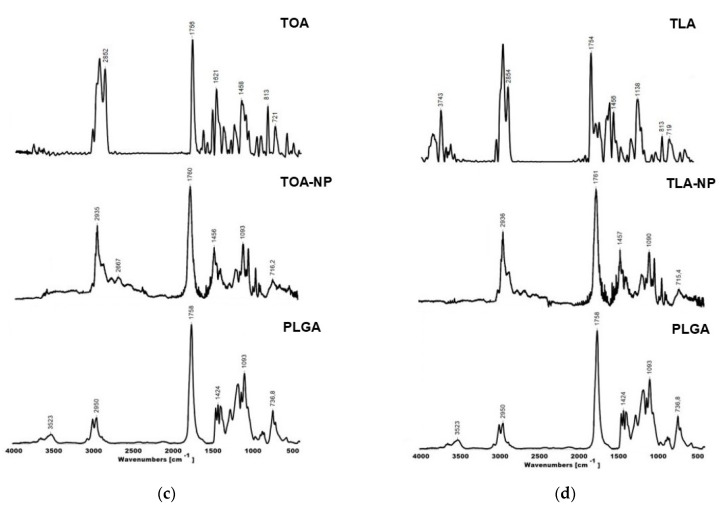
FTIR spectra of (**a**) OA-NPs and main components, (**b**) LA-NPs and main components, (**c**) TOA-NPs and main components, and (**d**) TLA-NPs and main components.

We analyzed chemical interactions between the drugs and the polymer by FTIR spectroscopy (Figure 5).

The OA and LA FTIR spectra agreed well with previously reported results [36]. The peaks around the 2925–2800 cm^−1^ region corresponded to C-H stretching vibrations of –CH_3_ and –CH_2_ groups. The large peak around 1708 cm^−1^ corresponded to C=O double-bond stretching vibration of carbonyl groups. Peaks of the 1470–1200 cm^−1^ region corresponded to CH bending of –CH_3_ and –CH_2_. The fingerprint region lay between 1250 and 700 cm^−1^ and corresponded to stretching vibration of C-O ester groups and CH_2_ vibration [37]. 

TOA and TLA showed similar results to OA and LA, with large peaks around 1760 cm^−1^ (C=O stretching). Ring vibration of thymol in TOA and TLA was at 813 cm^−1^. These bands were usually very intense in the FTIR spectra and could be attributed to out-of-plane CH wagging vibrations, which were the most significant signals used in distinguishing different types of aromatic ring substitutions [38]. Thymol in TOA and TLA showed bands in the range of 1600–1585 for C=C stretching vibration. Other key characteristic peaks of thymol in TOA and TLA were observed around the 700–1289 cm^−1^ region [39].

PLGA showed characteristic peaks at 3523 cm^−1^ (OH stretching), 2996–2950 cm^−1^ (C-H stretching), 1758 cm^−1^ (C=O stretching), 1424 and 1093 cm^−1^ (asymmetric and symmetric C-C(=O)-O stretching, respectively), and 900–700 cm^−1^ (C-H bending), which agreed with previously reported results [40].

TOA-NPs and TLA-NPs showed absorption bands in the 1760–1754 cm^−1^ region, corresponding to the stretching vibration of carbonyl groups in PLGA and substances. Bands of medium intensity in the 1455–1457 cm^−1^ region and in the 1093–1090 cm^−1^ region corresponded to the C-C(=O)-O stretches in PLGA. The TOA-NP and TLA-NP spectra lacked ring vibration of thymol in TOA and TLA at 813 cm^−1^, probably due to signal overlapping. The shift of the bands in the 2856–2960 cm^−1^ region (C-H valence vibrations) was caused by physical interaction between the drugs and the polymer matrix.

The spectra of nanoparticles lacked new absorption bands, indicating the absence of chemical interactions between the substances and the polymer and the formation of new chemical bonds.

### 3.7. In Vitro Drug Release Study

Earlier, we evaluated theoretical parameters for oral and intravenous administration routes of the designed formulations. Thus, we performed a release study in media simulating the GI tract (pH 1.2 and 6.8) and blood (pH 7.4).

**Figure 6:**
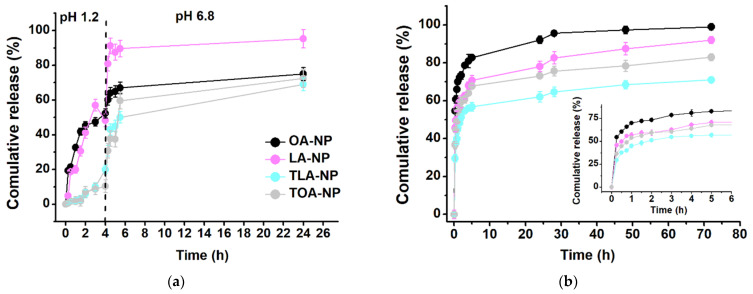
In vitro release of OA, LA, TLA, and TOA from nanoparticles in (**a**) SGF at pH 1.2 and in SIF at pH 6.8 and (**b**) 0.01 M PBS media at pH 7.4 (means ± SDs, *n* = 3).

Gastrointestinal transit time is highly variable and can range from several minutes to several hours, depending on age, body posture, gender, osmolarity, and food intake. However, the transit time in the small intestine, which is the longest part of the GI tract (approximately 6 m in length), is considered relatively constant—around 3–4 h [41]. Thus, we carried out a release study in simulated gastric fluid (SGF) media (pH 1.2) for 4 h, followed by incubation in simulated intestinal fluid (SIF) media (pH 6.8) for 20 h (Figure 6a).

LA-NPs and OA-NPs showed similar release profiles in the SGF, releasing 50% of LA and OA during the first 4 h. In the SIF, LA-NPs exhibited fast release, achieving almost complete LA release immediately after media replacement. In the case of OA-NPs, we observed slow release of OA—up to 70% during the remaining 20 h. The release rate of TOA-NPs was negligible (10.3%) up to 4 h, but rapidly increased up to OA-NP values when the pH was raised to 6.8. TLA-NPs showed a similar pattern, with a modest release rate in the SGF (10.7%) and rapid release in the SIF up to 64.7%.

The tested formulations exhibited similar release patterns, with slow release in the SGF and rapid release in the SIF, probably because of the acidic environment influence on the carboxyl groups of PLGA, leading to protonation and subsequent nanoparticle aggregation. This aggregation resulted in the formation of stable structures that restricted drug release. Contrastingly, the pH increment in the SIF triggered fast drug release, probably because water absorption was facilitated by the glycolic acid groups, inducing water penetration toward the core of the nanoparticles [42].

In the case of the 0.01M PBS media (pH 7.4), all the tested samples had a biphasic profile with initial burst release during the first 5 h, followed by a prolonged sustained phase up to 72 h (Figure 6b). The observed pattern is characteristic of the release of hydrophobic drugs from PLGA matrixes, occurring due to drug detachment from NP surfaces or the release of drug molecules located near the inner surfaces [43]. The OA-NPs exhibited the fastest initial release phase, with 83% for 5 h. Next, the LA-NPs and TOA-NPs revealed similar release rates: 71% of LA and 68% of TOA for 5 h. Finally, TLA-NPs showed the slowest burst release—55% for 5 h. We observed a strong relationship between DL and EE values and the release rates of the compounds. Thus, the highest DL value and the lowest EE value (17%) of OA-NPs indicated that a pronounced fraction of the compound was attached to the NP surfaces and, consequently, was released during the initial stages. Despite the TLA-NPs having the lowest DL value, their EE value was 93%, indicating that the main fraction was located inside the PLGA matrix and that only a negligible amount of the compound was attached to the NP surfaces. In this case, diffusion of the drug through the polymer matrix resulted in a slower release rate.

Next, we simulated the drug release kinetics of the tested compounds to determine the limiting phase of the process, which corresponded to the main mechanism of drug release from the polymer matrix. We selected five conventional models to describe the release kinetics of the intact and conjugated FAs (Table 5). Here, the adjusted coefficient of determination (R^2^ adj) was a criterion for fitting the experimental data to the release curve.

The Weibull model was best fitted for describing the release kinetics of the tested compounds in both types of media: for pH 1.2–6.8, the R^2^ adj values were >0.8017, and for pH 7.4, the R^2^ adj values were >0.9896. We evaluated the exponent of time (β) of the Weibull function to estimate the correlation between the exponent values and the possible transport of a drug through the polymer matrix (Table 6).

The value of the exponent (β) is an indicator of the mechanism of transport of a drug through a polymer matrix. Estimates for β ≤ 0.75 indicate Fickian diffusion, while anomalous and Case II transport are associated with β values in the range of 0.75 < β < 1. For values of β higher than 1, the drug transport follows a super Case II transport [44]. In the case of pH 1.2–6.8, besides OA, β values for all the formulations were higher than 1, which corresponded to super Case II transport. This type of transport indicated that the drug is mainly released through the erosion of the polymer matrix [45]. In the case of OA, the β value was <0.75, indicating that the release of OA was mainly limited by diffusion, while the polymer swelling influenced the release kinetics negligibly [46]. Interestingly, the difference in transport mechanisms between the formulations could be attributed to the OA-NPs having lower EE values compared to the other NPs. Fatty acids can exhibit porogen properties, making pores when extracted from a polymer matrix to a water phase [47]. Compared to non-porous particles, highly porous PLGA matrixes tend to uptake water more rapidly, resulting in fast degradation, which starts immediately when the polymer makes contact with water. Next, PLGA hydrolytic erosion occurs when the degradation starts and the first oligomers can diffuse out of the particles [48]. Thus, we assumed that high EE values in the LA, TOA, and TLA nanoformulations indicated faster pore formation, higher water uptake, and more rapid erosion, unlike in the case of the OA-NPs, where low EE values contributed to negligible erosion and pronounced diffusion through the pores.

Regarding pH 7.4, the tested formulations exhibited β values < 0.75, which corresponded to Fickian diffusion. This type of transport indicates that the release of compounds is mainly limited by diffusion, while the process of swelling of the polymer matrix has little effect on the release kinetics [49]. Our results agreed with previously reported data describing hydrophobic drug-release mechanisms [50].

Overall, the release kinetics study confirmed prolonged release of TOA-NPs and TLA-NPs of up to 72% and 65%, respectively, in 24 h in the GI tract simulation media and prolonged release of up to 57% and 68%, respectively, in 72 h in the blood simulation media. Modeling revealed that TOA-NP and TLA-NP release occurred in controlled mode corresponding to the Weibull model.

### 3.8. Antibacterial and Antifungal Efficacy

Next, we evaluated the antibacterial and antifungal properties of the free and encapsulated OA, LA, TOA, and TLA against the Gram-positive bacteria *B. cereus* and *S. aureus* and the Gram-negative bacterium *E. coli*, along with the fungi *C. albicans* and *A. niger*, by broth microdilution assay. We used thymol as a positive control, since it was selected as a phenolic moiety in TOA and TLA, and its antimicrobial activity has been widely confirmed in various studies [51]. The value of the minimum inhibitory concentration (MIC) corresponded to the first well in which the visible growth of microorganisms was lacking. We evaluated the activity of nanoparticles by the value of the minimum bactericidal concentration (MBC) because nanoparticle suspensions at high concentrations are opaque, making it impossible to assess clearly the growth of cultures.

MIC and MBC values for unmodified OA and LA against the studied microorganisms indicated negligible activity (Table 7 and Table 8), which agreed with previous results [52,53].

*B. cereus* was the most susceptible to both free and encapsulated OA and LA. Moreover, encapsulated OA and LA showed higher activity against *B. cereus* compared to free FAs. 

Unlike TOA and TOA-NPs, which lacked activity towards any of the test strains (MBC 15,000 μg/mL), TLA and TLA-NPs were active against *B. cereus*, probably due to the higher activity of the corresponding unmodified LA, which agreed with previous results [53]. Notably, TLA-NPs exhibited pronounced activity compared to free TLA. This beneficial effect could be attributed to the enhanced ability of TLA to permeabilize and depolarize the cytoplasmic membrane [51] and to the improved ability of NPs to penetrate cell membranes [1,54].

OA and LA showed similar effects against *C. albicans*, but OA had lower MIC values compared to LA for *A. niger*. Encapsulation resulted in an OA efficiency increment against *C. albicans* and enhanced LA activity against *A. niger*. The activity of TOA against *C. albicans* was comparable, with corresponding unmodified fatty acids. NP encapsulation did not increase TOA activity. In contrast, the MBC value for *A. niger* was lower for TOA-NPs, indicating the favorable effect of encapsulation on TOA activity. TLA revealed the most prominent activity against both fungal strains. Surprisingly, TLA-NPs showed lower efficacy compared to TLA, probably due to the insufficient release rate of TLA from NPs: fatty acids are located mainly within lipid bilayers, and the slow release rate potentially limits membrane integrity disruption [55].

An earlier study indicated the limited effect of thymol modification on antimicrobial efficacy—Sturabotti and co-authors reported that a hyaluronic acid-thymol conjugate had similar MIC values to intact thymol but exhibited better hydrophilicity [56].

Moreover, Arasoglu and co-authors emphasized that PLGA nanoparticles’ antimicrobial efficacy can be heavily influenced by the size and shape of the nanoparticles [57]. Therefore, a careful study of the physical properties of nanoparticles is necessary to ensure the uniformity of experimental conditions.

Overall, bioconjugation of OA and LA resulted in different outcomes. While TOA lacked activity against bacterial strains and showed a modest effect against *C. albicans*, TLA exhibited efficacy against Gram-positive bacteria and fungal strains. TLA encapsulation resulted in enhanced efficacy in the case of bacterial strains, but the lower MBC values for *C. albicans* and *A. niger* can probably be explained by the slow TLA release and the difference in the composition of the bacterial and fungal cell walls [58]. 

### 3.9. In Vitro Cytotoxicity

Modification of known anticancer drugs with fatty acids is a widely used strategy to enhance the efficacy of therapy. Tumor cells ingest fatty acids to use them as biochemical precursors and energy sources. Studies of docosahexaenoic acid and paclitaxel [59], oleic acid and chlorin e6 [60], and leuprolide acetate and lauric acid [61] revealed beneficial effects, like enhanced stability, cytotoxic efficacy, and pharmacokinetics.

We performed a cytotoxic activity study to determine the influence of the conjugation and encapsulation process on compound cytotoxicity. Doxorubicin was used as a positive control. Table 9 shows the cytotoxicity results of free and encapsulated OA, LA, TOA, and TLA against MCF-7, K562, and A549 cell lines. These cell lines were selected based on the prediction results of PASS.

Intact OA and LA exhibited modest activity against all tested cell lines, showing a negligible difference in cytotoxicity between free and encapsulated compounds. Our results agreed with previous reports [62,63]. Bioconjugation of OA and LA lacked a pronounced increment in activity compared to the corresponding intact fatty acids. Jóźwiak and co-authors observed that fatty acids can stimulate different responses in cancer cells, including stimulation of cell proliferation [10]. Thus, the lack of pronounced effects on cell cultures could be explained by the rapid metabolism of the studied compounds. In another study, the increased cytotoxic activity of FAs modified with cytarabine was explained by increased lipid solubility and transmembrane transport interference [64], providing efficient membrane penetration and drug accumulation. 

Apparently, the encapsulation of TOA and TLA provided similar effects, showing a significant increase in cytotoxicity against all tested cell lines. The enhanced activity of PLGA nanoparticles is probably explained by the highly efficient encapsulation of TOA and TLA and the prolonged release, which was favorable for drug accumulation in the cells. In contrast, the OA-NPs and LA-NPs had high DL and low EE values, which implies that significant fractions of the compounds were located on the NPs’ surfaces—the burst release of OA and LA from the NPs’ surfaces resulted in the floating of oily compounds in the cell culture media, reducing drug accumulation and cytotoxic effects. Moreover, the PLGA matrix served as a protective shell, preventing the fast metabolism of TOA and TLA in the cell culture media. 

Despite free and encapsulated compounds exhibiting lower efficacy compared to doxorubicin, the designed formulation revealed high potential. Our previous studies of anticancer and antibacterial PLGA nanoformulations confirmed significant tumor growth inhibition in animal models [20] and pronounced reductions in therapeutic doses of antibiotics in vivo [17]. Beneficial in vivo effects with modest in vitro results can be explained by (1) improved drug solubility and easier administration, (2) prolonged circulation time providing effective drug accumulation in target cells, and (3) enhanced bioavailability, which reduces the dose and, consequently, the toxicity of a drug [65,66].

Overall, encapsulation of TOA and TLA resulted in increased cytotoxic activity compared to TOA and TLA and corresponding fatty acids, and this is going to be clarified further in in vivo cancer models.

## 4. Conclusions

Based on PASS predictions and ADMET 2.0 simulations, we provided a rationale of OA and LA modification with biologically active phenol derivatives and further encapsulation into PLGA nanoparticles. UV-vis and FTIR studies confirmed the lack of fatty acid chemical interactions with PLGA matrixes after encapsulation. Modified OA and LA exhibited prolonged sustained release from NPs both in GI tract simulation media and blood simulation media. Notably, in the first case, we observed that the main fraction of the encapsulated compounds was released at pH 6.8, corresponding to intestinal conditions, where microbiota are concentrated. Kinetics analysis revealed the best-fitted model—the Weibull model limited by Fickian diffusion. Comparing TOA-NPs and TLA-NPs, we observed that the latter showed higher activity against both bacterial and fungal strains. TLA-NPs showed higher activity against Gram-positive bacteria in contrast with TLA. However, we observed the opposite effect against fungal strains. Finally, the designed PLGA nanoparticles loaded with TOA and TLA showed high cytotoxicity compared to unmodified compounds. This work shows the promise of PLGA nanoparticles loaded with modified fatty acids in various biomedical applications, including antimicrobial efficacy and anticancer treatment.

## Figures and Tables

**Table 1:** Results of antibacterial activity prediction.

Confidence	Bacterial Strain	Confidence	Bacterial Strain
TOA		TLA	
0.506	*Bacteroides stercoris* (G −ve) ^1^	0.588	*Staphylococcus simulans* (G+ve)
0.452	*Streptococcus viridans* (G +ve) ^2^	0.479	*Bacteroides stercoris* (G −ve)
0.404	*Staphylococcus simulans* (G +ve)	0.418	*Streptococcus viridans* (G +ve)
0.373	*Clostridium cadaveris* (G +ve)	0.351	*Clostridium cadaveris* (G +ve)
0.367	*Lactobacillus plantarum* (G +ve)	0.349	*Lactobacillus plantarum* (G +ve)
OA		LA	
0.512	*Streptococcus viridans* (G +ve)	0.718	*Staphylococcus saprophyticus* (G +ve)
0.486	*Staphylococcus simulans* (G +ve)	0.670	*Staphylococcus simulans* (G +ve)
0.411	*Lactobacillus plantarum* (G +ve)	0.414	*Streptococcus viridans* (G +ve)
0.391	*Salmonella enteritidis* (G +ve)	0.389	*Lactobacillus plantarum* (G +ve)
0.385	*Listeria innocua* (G +ve)	0.366	*Streptococcus mutans* (G +ve)

^1^ G −ve—*Gram-negative bacteria;* ^2^ G +ve—*Gram-positive bacteria.*

**Table 2:** Results of anticancer activity prediction.

Pa	Pi	Cell Line	Cell Line (Full Name)	Tissue	Tumor Type
TOA					
0.629	0.004	SK-MEL-1	Metastatic melanoma	Skin	Melanoma
0.569	0.024	NCI-H838	Non-small-cell lung cancer, stage 3	Lung	Carcinoma
0.502	0.038	DMS-114	Lung carcinoma	Lung	Carcinoma
TLA					
0.649	0.003	SK-MEL-1	Metastatic melanoma	Skin	Melanoma
0.560	0.026	NCI-H838	Non-small-cell lung cancer, stage 3	Lung	Carcinoma
0.526	0.028	DMS-114	Lung carcinoma	Lung	Carcinoma
OA					
0.583	0.004	A2058	Melanoma	Skin	Melanoma
0.563	0.011	SK-MEL-1	Metastatic melanoma	Skin	Melanoma
0.551	0.029	NCI-H838	Non-small-cell lung cancer, stage 3	Lung	Carcinoma
0.543	0.022	DMS-114	Lung carcinoma	Lung	Carcinoma
0.508	0.018	IGROV-1	Ovarian adenocarcinoma	Ovarium	Adenocarcinoma
LA					
0.607	0.009	DMS-114	Lung carcinoma	Lung	Carcinoma
0.595	0.005	SK-MEL-1	Metastatic melanoma	Skin	Melanoma
0.544	0.006	A2058	Melanoma	Skin	Melanoma
0.513	0.043	NCI-H838	Non-small-cell lung cancer, stage 3	Lung	Carcinoma

**Table 3:** ADMETlab 2.0 prediction results.

	Molecular Weight	Flexibility	LogP	F_30%_ ^1^	PPB ^2^, %	Vd ^3^, L/kg	Metabolism	T1/2 ^4^
TOA	414.31	2.25	9.31	0.9–1.0 (poor)	100	2.71	CYP2C9 substrate	0.154
TLA	412.33	1.89	8.50	0.9–1.0 (poor)	101	2.07	CYP2C9 substrate	0.408
OA	282.26	7.50	6.17	0.9–1.0 (poor)	99	0.78	CYP2C9 substrate	0.811
LA	280.24	4.67	4.60	0.9–1.0 (poor)	99	0.63	CYP2C9 substrate	0.875

^1^ F_30%_—30% bioavailability; ^2^ PPB—plasma protein binding; ^3^ Vd—volume of distribution; ^4^ T1/2—half-life.

**Table 4:** The influence of batch size on parameters of nanoparticles loaded with OA, LA, TOA, and TLA. Data shown as means ± SDs (*n* = 3).

Lab Scale	Size, nm	PDI	Zeta, mV	DL, %	EE, %
LA-NPs					
1-fold	263 ± 2	0.261 ± 0.031	−7.2 ± 1.0	26 ± 0.2	58 ± 3
**4-fold**	**224 ± 1**	**0.233 ± 0.032**	**−17.4 ± 1.4**	**24 ± 0.3**	**60 ± 2**
8-fold	241 ± 1	0.272 ± 0.025	−14.5 ± 2.0	24 ± 0.4	54 ± 2
12-fold	267 ± 3	0.282 ± 0.022	−10.8 ± 1.6	28 ± 0.3	29 ± 4
OA-NPs					
1-fold	207 ± 1	0.099 ± 0.010	−12.6 ± 1.8	28.1 ± 0.1	14 ± 1
**4-fold**	**222 ± 1**	**0.143 ± 0.012**	**−13.1 ± 1.4**	**26.0 ± 0.2**	**17 ± 2**
8-fold	245 ± 2	0.116 ± 0.021	−10.9 ± 1.5	18.5 ± 0.2	16 ± 3
12-fold	215 ± 2	0.210 ± 0.029	−11.8 ± 2.1	21.3 ± 0.3	13 ± 3
TLA-NPs					
1-fold	243 ± 3	0.212 ± 0.020	−10.8 ± 3.8	8.1 ± 0.4	86 ± 2
4-fold	222 ± 1	0.243 ± 0.012	−12.8 ± 1.4	9.2 ± 0.2	88 ± 2
8-fold	287 ± 3	0.235 ± 0.031	−14.6 ± 1.8	8.0 ± 0.3	81 ± 1
**12-fold**	**227 ± 3**	**0.208 ± 0.019**	**−11.2 ± 2.6**	**8.9 ± 0.3**	**93 ± 4**
TOA-NPs					
1-fold	282 ± 2	0.289 ± 0.038	−7.2 ± 1.1	18.7 ± 0.2	83 ± 3
4-fold	224 ± 1	0.227 ± 0.012	−17.6 ± 1.4	13.4 ± 0.2	77 ± 3
8-fold	267 ± 3	0.282 ± 0.025	−14.5 ± 1.0	15.4 ± 0.2	64 ± 2
**12-fold**	**212 ± 2**	**0.180 ± 0.015**	**−10.4 ± 1.6**	**16.5 ± 0.3**	**71 ± 3**

**Table 5:** Model fitting of in vitro release kinetics of tested compounds.

Media	Sample	Mathematical Model				
		Zero Order	First Order	Higuchi	H-C ^1^	Weibull
pH 1.2–6.8 (gastrointestinal tract)	OA-NP	−1.4674	0.7613	0.5211	0.6235	**0.9506**
	LA-NP	−0.3605	0.9214	0.6710	0.8276	**0.9252**
	TOA-NP	0.6000	0.7980	0.7159	0.7696	**0.8271**
	TLA-NP	0.4020	0.7412	0.7112	0.7780	**0.8017**
pH 7.4 (blood)	OA-NP	−4.6362	0.6894	−1.9383	−2.8339	**0.9980**
	LA-NP	−4.5278	0.4694	−1.8589	−2.6963	**0.9971**
	TOA-NP	−3.7303	0.1173	−1.3627	−2.2060	**0.9946**
	TLA-NP	−3.5261	−0.6163	−1.2422	−2.3814	**0.9896**

^1^ H-C—Hixson–Crowell model.

**Table 6:** Calculation of Weibull exponent (β).

Media	OA-NP	LA-NP	TOA-NP	TLA-NP
pH 1.2–6.8	0.418	1.863	3.270	2.631
pH 7.4	0.275	0.220	0.182	0.160

**Table 7:** Antibacterial efficacy results of tested compounds and nanoformulations.

Test Samples	*E. coli* ATCC 25922		*B. cereus* ATCC 10702		*S. aureus* ATCC 29213	
	MIC, µg/mL	MBC, µg/mL	MIC, µg/mL	MBC, µg/mL	MIC, µg/mL	MBC, µg/mL
Thymol	15,000	15,000	937.5	937.5	937.5	937.5
OA	15,000	15,000	937.5–468.75	1875	15,000	15,000
OA-NP	ND ^1^	15,000	ND	468.75	ND	3750
LA	15,000	15,000	3750–1875	3750	15,000	15,000
LA-NP	ND	15,000	ND	468.75	ND	15,000
TOA	15,000	15,000	15,000	15,000	15,000	15,000
TOA-NP	ND	15,000	ND	15,000	ND	15,000
TLA	15,000	15,000	1875	1875	1875	15,000
TLA-NP	ND	15,000	ND	468.75	ND	3750

^1^ ND—not detected.

**Table 8:** Antifungal efficacy results of tested compounds and nanoformulations.

Test Samples	*Candida albicans* ATCC 10231		*Aspergillus niger* 37a	
	MIC, µg/mL	MBC, µg/mL	MIC, µg/mL	MBC, µg/mL
Thymol	234.38	234.38	234.38	234.38
OA	7500	15,000	937.5–468.75	468.75
OA-NP	ND ^1^	1875	ND	15,000
LA	7500	15,000	7500	15,000
LA-NP	ND	15,000	ND	468.75
TOA	7500	15,000	15,000	15,000
TOA-NP	ND	15,000	ND	3750
TLA	468.75	937.5	234.38	1875
TLA-NP	ND	7500	ND	15,000

^1^ ND—not detected.

**Table 9:** Cytotoxicity results of intact and bioconjugated fatty acids before and after encapsulation.

Sample	IC_50_, µM		
	MCF-7	K562	A549
Doxorubicin	34.3	7.9	4.6
OA	1390	731	487
OA-NP	1263	694	349
LA	796.2	498	986.3
LA-NP	490	372	766.8
TOA	1520	1328.4	657
TOA-NP	94.5	112.2	205
TLA	1103	1246	754
TLA-NP	39.0	25.2	189

## Data Availability

Data are contained within the article.

## Supplementary Materials

The following supporting information can be downloaded at: https://www.mdpi.com/article/10.3390/polym16010072/s1, Figure S1: ^1^H NMR spectrum of 2-isopropyl-5-methylphenyloleate (TOA); Figure S2: ^1^H NMR spectrum of 2-isopropyl-5-methylphenyllinoleate (TLA); Figure S3: ^13^C NMR spectrum of 2-isopropyl-5-methylphenyloleate (TOA); Figure S4: ^13^С NMR spectrum of 2-isopropyl-5-methylphenyllinoleate (TLA).
