# Supplementary material for: Thymol-Modified Oleic and Linoleic Acids Encapsulated in Polymeric Nanoparticles: Enhanced Bioactivity, Stability, and Biomedical Potential
Source: Polymers (Basel). 2023 Dec 26;16(1):72. doi: 10.3390/polym16010072 (PMC10781094; doi:10.3390/polym16010072)
Supplement: Supplementary file 1 [file polymers-16-00072-s001.zip › polymers-2764915-supplementary.pdf]

Supplementary

Thymol-Modified Oleic and Linoleic Acids Encapsulated in Polymeric Nanoparticles: Enhanced Bioactivity, Stability, and Biomedical Potential

Maria B. Sokol[1,*], Vera A. Sokhraneva[2,3], Nataliya V. Groza[2], Mariia R. Mollaeva[1], Nikita G. Yabbarov[1], Margarita V. Chirkina[1], Anna A. Trufanova[1], Vladimir I. Popenko[3], Elena D. Nikolskaya[1,*]

1 N.M. Emanuel Institute of biochemical physics of Russian academy of sciences, 119334, Moscow, Russia; mollaevamariia@gmail.com (M.R.M.); yabbarovng@gmail.com (N.G.Y.); chir.marg@mail.ru (M.V.C.); puzinoaa@gmail.com (A.A.T.)

2 N.A. Preobrazhensky Department of Chemistry and Technology of Biologically Active Compounds, Medicinal and Organic Chemistry, M.V. Lomonosov Institute of Fine Chemical Technologies, MIREA – Russian Technological University, 119571 Moscow, Russia; sokhraneva.v@mail.ru (V.A.S.); grozanv@gmail.com (N.V.G.)

3 Engelhardt Institute of Molecular Biology, Russian Academy of Sciences, 11999, Moscow, Russia; popenko@eimb.ru (V.I.P.)

* Correspondence: mariyabsokol@gmail.com (M.B.S.); elenanikolskaja@gmail.com (E.D.N.)

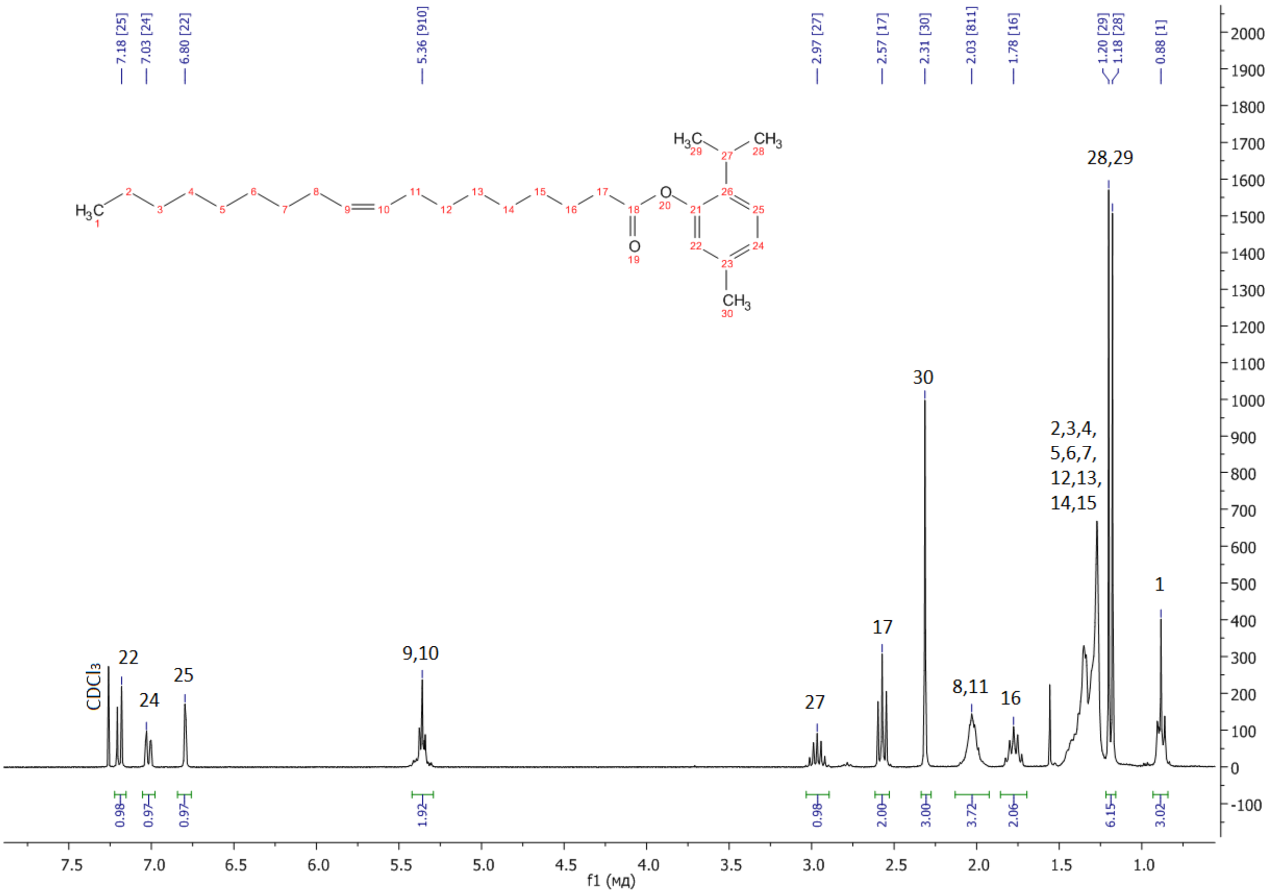

Figure S1. ^{1}H NMR spectrum of 2-isopropyl-5-methylphenyloleate (TOA).

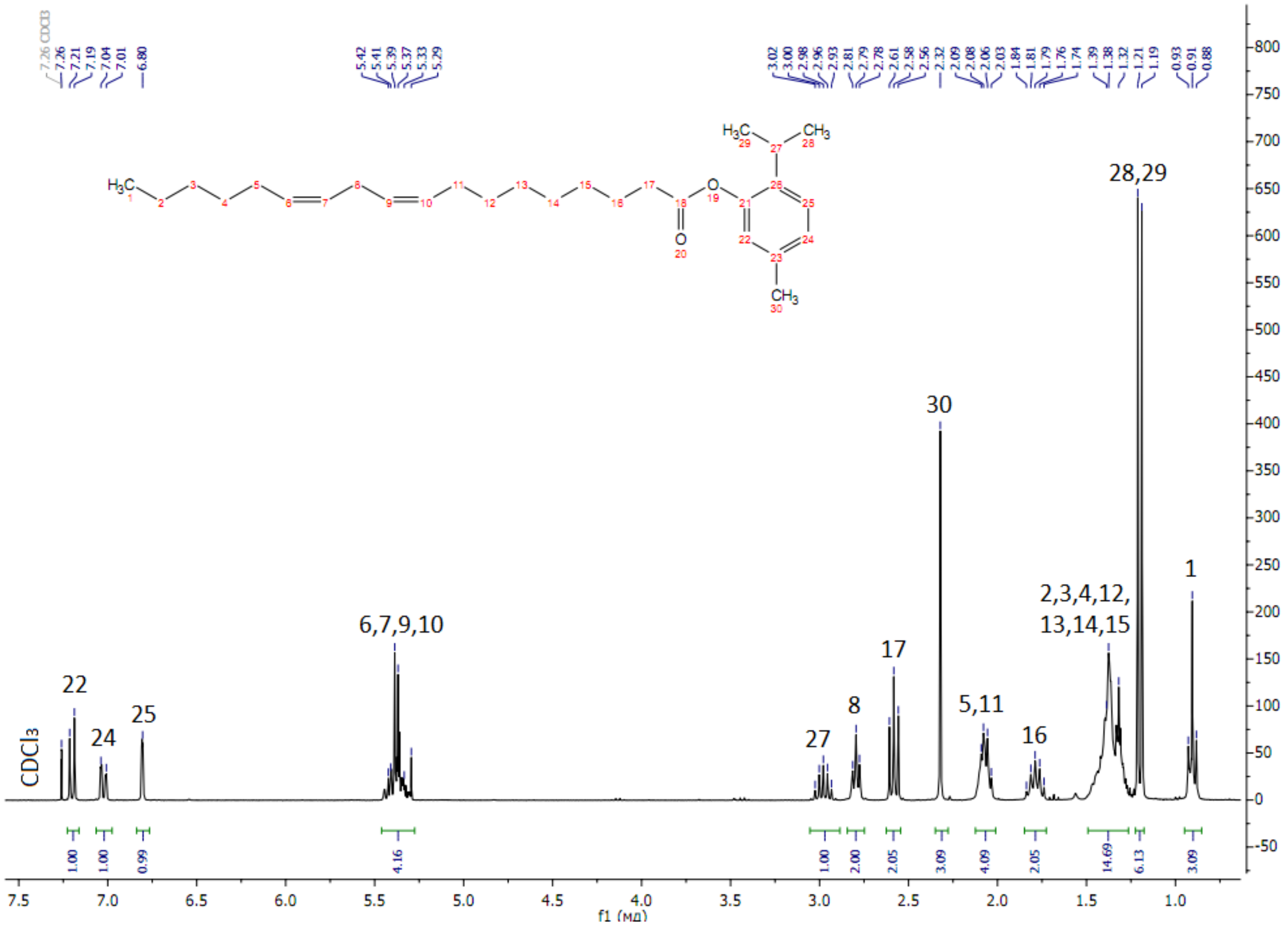

Figure S2. ^{1}H NMR spectrum of 2-isopropyl-5-methylphenyllinoleate (TLA).

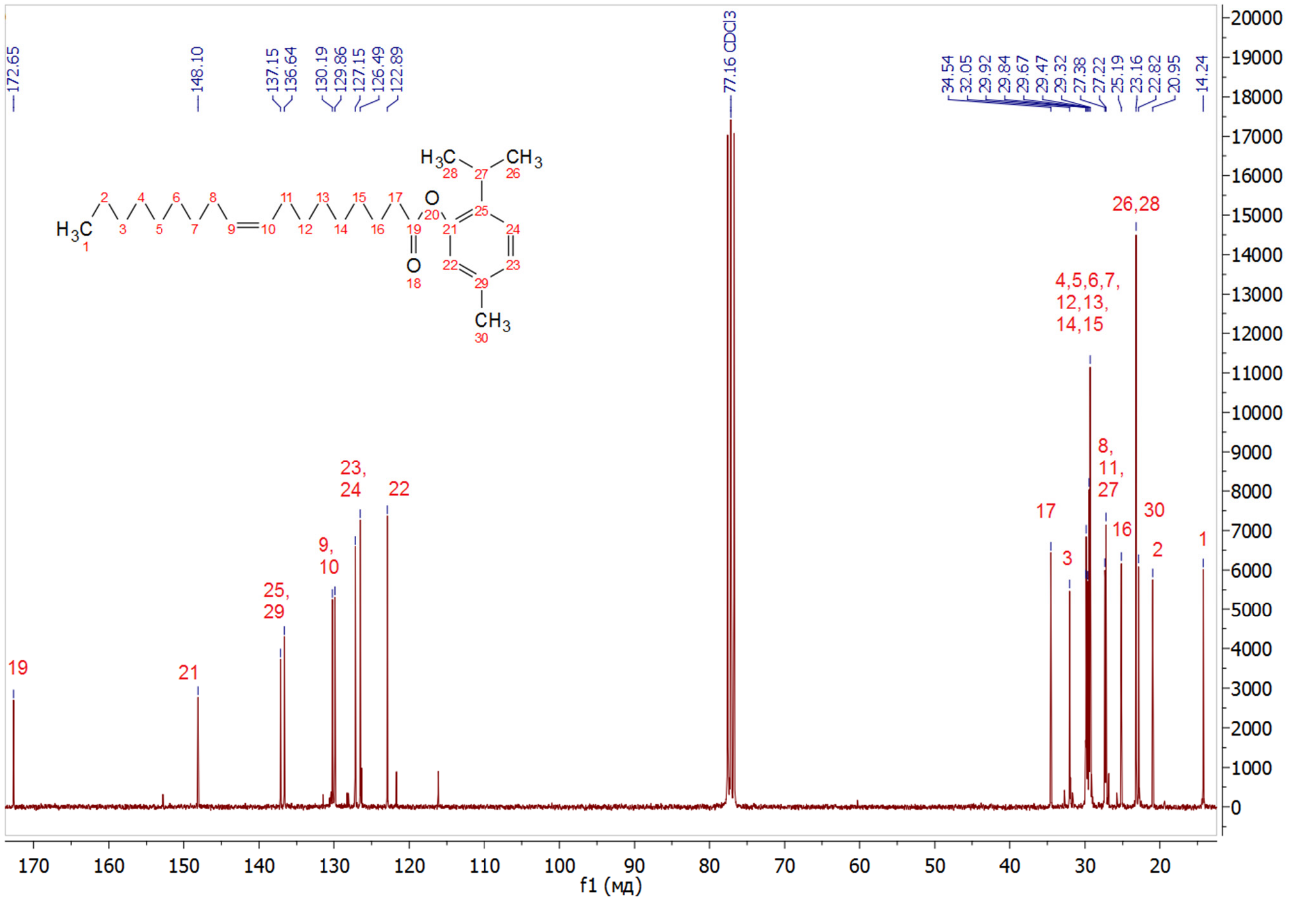

Figure S3. ^{13}C NMR spectrum of 2-isopropyl-5-methylphenyloleate (TOA).

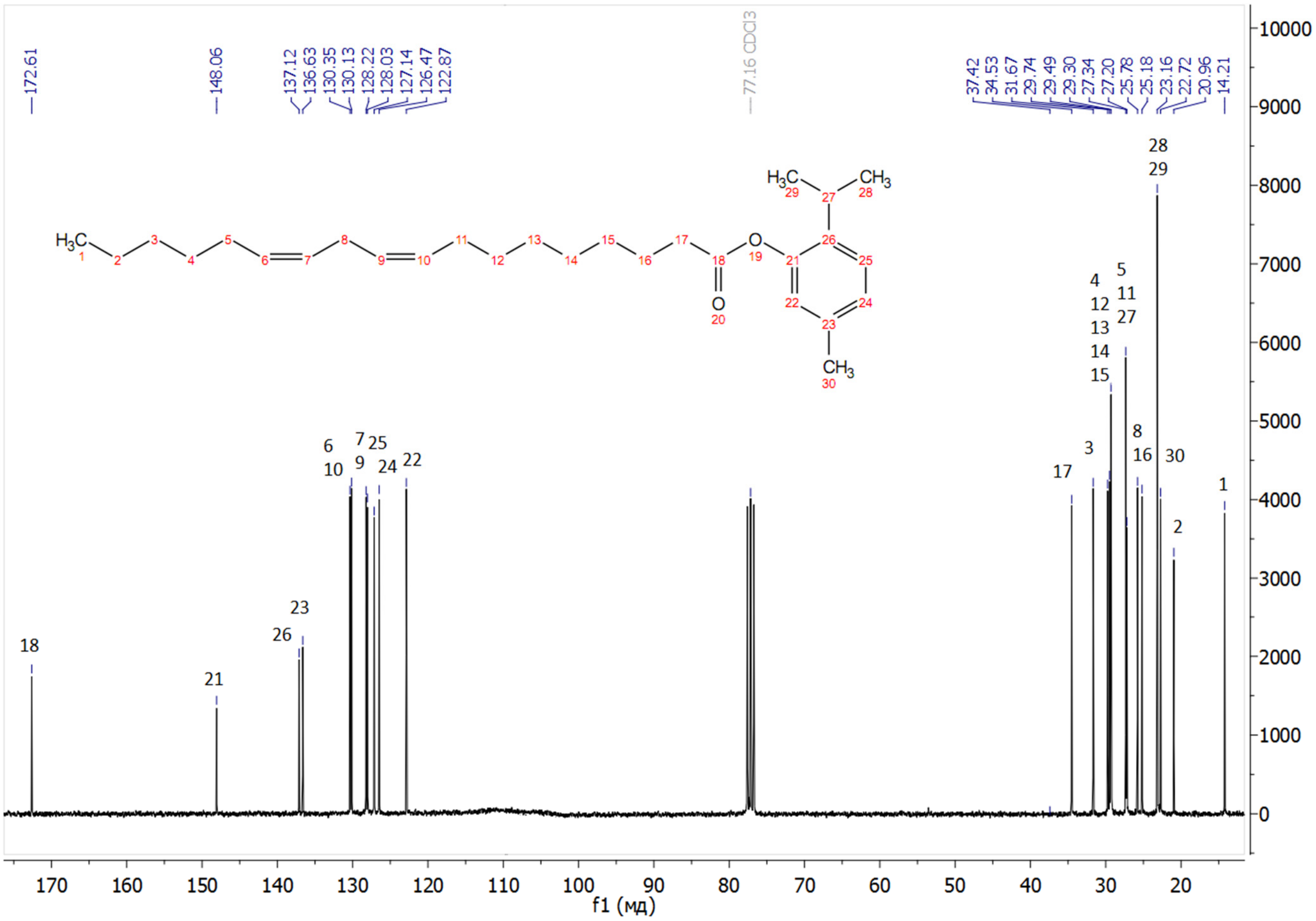

Figure S4. ^{13}C NMR spectrum of 2-isopropyl-5-methylphenyllinoleate (TLA).
